# Refugee Women with a History of Trauma: Gender Vulnerability in Relation to Post-Traumatic Stress Disorder

**DOI:** 10.3390/ijerph18094806

**Published:** 2021-04-30

**Authors:** Macarena Vallejo-Martín, Ana Sánchez Sancha, Jesús M. Canto

**Affiliations:** 1 Department of Social Psychology, Social Work, Social Anthropology and East Asia Studies, Faculty of Psychology and Speech Therapy, University of Malaga, 29016 Malaga, Spain; jcanto@uma.es; 2 Department of Personality, Assessment and Psychological Treatment, Faculty of Psychology and Speech Therapy, University of Malaga, 29016 Malaga, Spain; sanchezsanchaana@gmail.com

**Keywords:** refugee women, post-traumatic stress disorder (PTSD), traumatic experiences, sexual violence, systematic review

## Abstract

Refugees represent a population whose living conditions have a strong impact on their mental health. High rates of post-traumatic stress disorder (PTSD), more than other mental disorders, have been found in this group, with women having the highest incidence. The objective of the present systematic review was to identify and examine studies from the last fifteen years on the relationship between the impact of traumatic experiences and PTSD psychopathology in refugee women. Twelve studies were included, from which the overall results approved this relation. In addition, six of these studies show that exposure to sexual trauma in refugee women is associated with the high odds of being at risk for PTSD. These findings suggest that gender-related traumatic experiences can explain the high rate of PTSD in refugee women and highlight the unmet need for psychosocial health care in this population.

## 1. Introduction

At present, we are in the midst of a humanitarian crisis that is causing millions of people to be displaced, for reasons of war, violence and the precarious situations in their countries of origin. Specifically, the UNHCR [1] estimates that 70.8 million people are forcefully displaced all over the world, resulting in 25.9 million refugees, half of whom are under the age of 18. The circumstances in which refugees often flee cause them to experience mental health problems and a significant deterioration in their psychological well-being [2,3]. Mental disorders and psychosocial problems are much more frequent in individuals that have had to confront these types of adversities, such as being exposed to a humanitarian crisis [4] or experiencing different types of discrimination [5]. In this sense, it was confirmed that refugees have a rate of mental disorders that is twice than that identified in migrant workers [6].

The traumatic events experienced before and during displacement cause refugees to suffer from psychological manifestations related to loss of persons or places with symptoms of grief, traumatic reactions and even dissociative symptoms or acute stress disorders [7]. Situations of uncertainty and problems of adaptation also arise, since refugees grapple with unpredictable and difficult changes [1]. As a consequence of this exposure to stressful and traumatizing factors, the majority of uprooted persons experience suffering and diverse emotional problems [8]. Among the different disorders and mental health problems that can affect refugees, post-traumatic stress disorder (PTSD) and depression are the most frequent [9,10,11].

### 1.1. Post-Traumatic Stress Disorder in Refugees

PTSD is considered to be the mental disorder most specific to refugees and it is associated with circumstances involving political and social repression, war and armed conflict, as well as violence and torture, to which they are subjected in their countries of origin [12]. Although post-traumatic stress symptoms can be different in diverse cultures, there is abundant historical and transcultural evidence indicating that exposure to extreme traumatic experiences can activate extreme psychological stress [13]. When individuals suffer from a traumatic experience and do not have the capacity to integrate it by themselves with their cognitive and emotional schemes, a dissociation mechanism may appear as a defense strategy, allowing them to continue living with their previous mental schemes while wiping out the painful part of the experience from their consciousness [14]. This disassociation mechanism can be understood as “a memory phobia” that prevents integration of traumatic events and disassociates these memories from the consciousness [15]. The continued use of disassociation as a way of coping with stress interferes with memory and psychological functioning, hindering the integration of associated memories and causing an inability to provide a coherent narrative of events [16,17]. Furthermore, sharing these negative experiences can cause shame, guilt and a high degree of distress, which very significantly impacts self-image and sense of personal worth, leading to a tendency to avoid and repress these issues [18].

The most widely known consequence of exposure to traumatic events is PTSD. PTSD was officially categorized as a mental disorder in the 1980 edition of the Diagnostic and Statistical Manual of Mental Disorder (DSM) [19]. An individual is diagnosed as suffering from PTSD when the symptoms caused by the trauma are severe and prolonged and interfere with their social and/or occupational functioning [20]. In the DSM-5 [21], PTSD is characterized by the presence of multiple symptoms that can be grouped into four clusters: (1) intrusive symptoms (for example, nightmares), (2) persistent avoidance of stimuli associated with the trauma, (3) negative alterations in cognition and mood associated with the traumatic event (for example, difficulty concentrating, guilt, etc.), and (4) alterations in arousal and reactivation associated with the traumatic event (for example, difficulty sleeping).

According to Weinstein, Khabbaz and Legate [22] “becoming a refugee is a powerful risk factor for indicators of psychological disorders such as stress, generalized stress and posttraumatic stress disorder (PTSD)”. Between 10 and 40% of refugees suffer from mental disorders after having experienced grave traumatic events in their countries of origin [9]. At the same time, different studies [11,23] have determined that among refugees, the rate of PTSD is higher than any other mental disorder. In addition, this group is ten times more likely to experience PTSD than the general population [24]. In the same vein, the study by Priebe, Giacco, and El-Nagib [25] points to a higher prevalence of PTSD and depression among refugees compared with the general population. Furthermore, refugees that have been submitted to torture and/or rape have the highest rates of PTSD [26]. For example, in a research on refugees from North Korea, significantly higher rates of suicidal thoughts and alcohol consumption after experiencing rape were found in comparison with refugees who had not undergone such types of traumatic experiences [27].

### 1.2. Gender Differences and PTSD: Traumatic Experiences in Refugee Women

According to Tolin and Foa [28], women are twice as likely as men to meet the criteria for PTSD, despite the fact that men usually experience greater overall exposure to traumatic events throughout their lifetimes. For Griffin, Resick and Mechanic [29], the dissociative response seems to be more common in individuals who have suffered sexual aggression. Along these lines, Norris [30] points out the survivors of rape are more prone to manifesting PTSD than victims of car accidents, robbery, physical assault, combat, fire, events involving deaths, and natural disasters. As such, it can be said that rape is the event most strongly linked to PTSD [31,32].

Wolfe and Kimerling [33] observed that the high incidence of sexual assault among women and the extremely high rates of PTSD in survivors of sexual assault contribute to the idea that being female is a risk factor for PTSD. According to different studies [30,31,34], throughout their lives, women contend with sexual assault and rape to a greater degree than men do. Accordingly, the most gender-based type of trauma is sexual violence. This gender difference in the risk of sexual victimization has clear implications for PTSD [35]. As such, in any study analyzing differences between men and women and PTSD, a significant role should be given to sexual-based trauma, since it is strongly linked to gender [35].

According to the UNHCR [36], refugee women often experience gender-based trauma, described as sexual violence that includes rape, forced impregnation, forced abortion, sexual trafficking, sexual slavery and the intentional spreading of sexually transmitted diseases, including HIV. Along these lines, Ward and Vann [37] establish that displaced women and girls are vulnerable to suffering from sexual violence, including forced sex/rape, sexual abuse by an intimate partner, child sexual abuse, coerced sex and sex trafficking in settings of humanitarian conflicts. For that reason, this population group presents a profile that is especially harmed and vulnerable and that often display symptoms of complex trauma [38]. However, studies focusing on the mental health status of refugee women are scarce [39]. This fact could be due to different reasons. First, upon interviewing extremely traumatized women, there is a serious risk that the questions may act to trigger traumatic content, which could be destabilizing, causing retraumatization and hindering recovery [40]. On the other hand, highly traumatized individuals can have difficulties concentrating long enough so as to complete extensive questionnaires [41]. At the same time, the language barrier can represent another obstacle to communicating with these individuals [42]. Additionally, women who have been the object of sexual assault might be rejected by their community and family and on many occasions, they are not able to make any sort of revelations in their interviews for asylum [43]. Likewise, in many cultures, rape and sexual assault are taboo subjects, for example, in Somali culture [44].

The aforementioned factors may be some of the reasons why few studies have examined specific violence in refugee women (suffered both in the fleeing phases in their countries of origin and in the experiences in the country of asylum where violence may persist) [45] and the effects it has on their mental health. The objective of this study is to present a systematic review that analyzes the relation between the impact of traumatic experiences in refugee women and developing PTSD. We consider this comprehensive review of the available research to date is original, innovative, and essential to understanding trauma in refugee women. Moreover, it is important for several reasons. Firstly, because of the need for attention that the refugee and asylum-seeking population have with respect to their mental health, which in most cases is affected by the experiences they have undergone. Secondly, it is essential to carry out this analysis from a gender perspective, determining the role that certain specific factors or traumatic events suffered by women, such as sexual violence understood in a broad sense, have on the deterioration of their psychological well-being and in the triggering of different disorders. Thirdly, so that the conclusions drawn from this study can be incorporated into resettlement or community integration programs for refugees, especially women, giving mental health a special relevance for the reconstruction of their lives.

## 2. Method

### Literature Search and Study Selection

A systematic review of published studies on the incidence of PTSD in refugee women was made. This search was carried out through four research platforms: PsycInfo, Pubmed, Science Direct and Scopus, being limited to articles published between April 2005 and December 2020. In order to obtain a specific search, result from the terms “post-traumatic stress” and “female refugees” or “refugee women” were used. We followed the Preferred Reporting Items for Systematic Reviews and Meta-Analysis (PRISMA) guidelines for our study search and selection [46,47].

With the purpose of establishing uniform selection standards, the following inclusion criteria were applied: (a) publications of studies and clinical trials; (b) type of study: cohort studies or case-control studies; (c) language: Spanish or English; (d) population group: refugee women with a history of trauma; (e) diagnosis: PTSD by means of internationally accepted assessments instruments, including interviews that apply criteria from the Diagnostic and Statistical Manual of Mental Health Disorders (DSM) or the International Classification for Diseases (CIE); (f) published during the period 2005–2020; (g) journals with a high impact factor indexed in the Journal Citation Reports (JCR). 

In addition, the following exclusion criteria were established: (a) narrative accounts, qualitative systematic reviews, and case studies; (b) subjects without a past history of trauma or PTSD diagnosis; (c) studies that used non-standardized tests; (d) studies whose data duplicates another study or that are not original; (e) articles in a language other than Spanish or English; (f) studies prior to 2005; (g) journals not having a high impact factor or not JCR-indexed.

**Figure 1:**
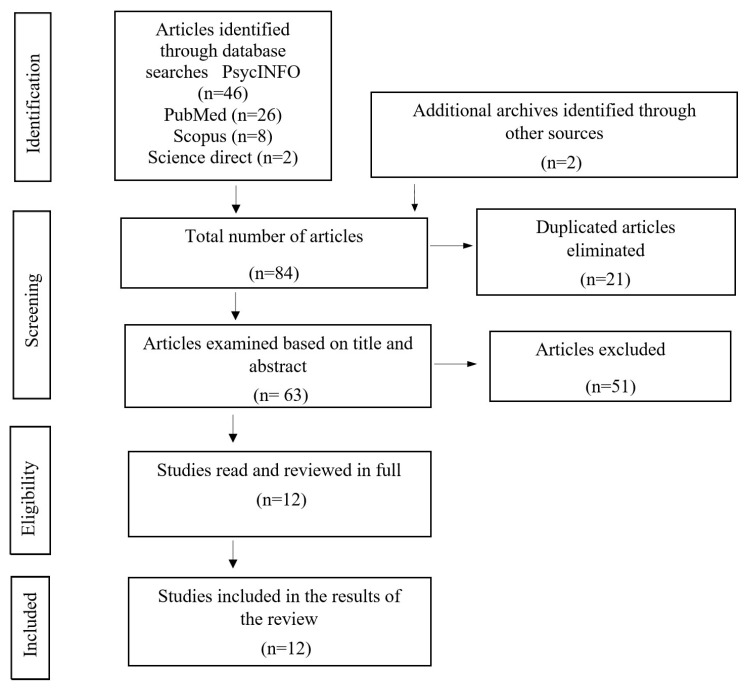
Preferred Reporting Items for Systematic Reviews and Meta-Analysis (PRISMA) flow diagram of each stage of the study selection.

The database search generated 82 results, which were classified in the Mendeley Desktop program. Two additional studies were identified, after reviewing articles from the bibliographic references of the research studies chosen. A total of 21 duplicated articles were excluded, so 63 articles were revised by title and abstract. Of these, 51 were excluded for not meeting the inclusion criteria, obtaining 12 articles that were read in their entirety and which were all chosen to be included in the results of our review. The search strategy results are summarized in Figure 1.

Unfortunately, a meta-analysis was not feasible given the diversity of the methodology used in the studies and the different co-variables dealt with in each one of them [48]. Accordingly, a narrative analysis was used, which enabled us to improve our understanding of PTSD in refugee women and help us to identify issues of importance to take into account in future paths for socio-healthcare intervention in this group. 

## 3. Results

A critical reading was performed and a synthesis made of the 12 selected articles. These articles provided original data on PTSD in refugee women. Table 1 shows the main results of the studies considered. 

In general, the chosen studies include participants from different countries, especially those who are in a situation of displacement (highlighting the Democratic Republic of the Congo and Somalia). The studies were carried out among adolescents and adults in different countries: The United States of America (1, 3), Canada (2), the Democratic Republic of the Congo (4, 5), the Republic of Uganda (6, 7), Germany (8, 11), Turkey (9), Australia (10), and the Republic of South Africa (12). Participants included both refugees and asylum seekers. The age of participants ranged between 13 and 85. The research was of a transversal nature, with the exception of study 3, for which a longitudinal follow-up took place at three and a half years of resettlement.

All of the studies evaluated PTSD, except number 11, which based its research on factors associated with the history of trauma measured by a questionnaire designed by psychologists and psychiatrists. In addition, some of the studies assessed dissociative symptoms (5, 8), sexual violence (4), sexual and/or physical violence (7), the impact of gender on traumatic experiences (10) and trauma and torture (1). With respect to sexual violence, the different studies operationalized it in a broad sense, including: forced marriages, individual and gang rapes, molestation, abuse, forced to undress, sexual slavery, forced to perform sexual acts with another person, stripped of clothing, forced abortions and touching.

### 3.1. PTSD and Other Mental Health Problems in Refugee Women

Of the twelve selected articles, five of them based their sample solely on female refugees (2, 5, 7, 8, and 11) and four specifically reported data regarding the prevalence of PTSD in this population group. Three of them showed similar and significant results in relation to the presence of PTSD symptoms in refugee women, with the percentage ranging from 66% to 71% [50,55,56], only 25.9% of the women had a score that indicated the presence of PTSD. At the same time, study 11 [42] was the only one that did not assess PTSD itself, but rather psychological symptoms through scores given by healthcare professionals. The most noteworthy symptoms related to trauma were nightmares, followed by insomnia and depression. The subjects also suffered from somatic ailments such as aches and pains, dizziness, and gastrointestinal issues.

In different studies (4, 5, 7 and 8) PTSD in refugee women was linked with other mental health problems, such as dissociative symptoms and depression. In Study 5, disassociation was pointed out as a predictor of PTSD severity in women with traumatic experiences, since the female participants that suffered from greater disassociation had a higher PTSD score. At the same time, study 8 [56] revealed that women patients with a higher degree of PTSD displayed greater disassociation and this correlated with symptoms of depression. Specifically, in this study, it was shown that the severity of PTSD in refugee women was linked to disassociation and severity of depression, being very closely interrelated. Other research studies also pointed to the relationship between depression and PTSD. For example, in study 5 [53] and study 7 [55] there was a high prevalence of depression (almost 95%), and in study 4 [52] it was observed that 40.5% of the sample met the criteria of having a major depressive disorder.

Study 1 [49] carried out a comparative analysis among three groups of women: women without children, women who had between one and six children and women with more than six children. It was found that the female refugees who were mothers with more than six children had undergone a higher number of torture and traumatic events, for which they had much more significant scores in PTSD than women who had fewer than six children or none. They also had higher rates of illiteracy, limited knowledge of spoken English, were caring for the children on their own, and were less likely to be working. At the same time, almost half of the women reported taking medication to calm themselves down, and nearly one in ten reported having seen a physician for their health problems. The majority used prayer to combat stress. The accounts involving greater exposure to trauma were highly correlated with more social, psychological and physical problems, and higher PTSD scores. Furthermore, 89% of women with large families reported having had to do things in order to survive that still bothered them, and almost two thirds (64%) of these women stated that they had been exposed to various types of torture, including rape.

### 3.2. Differences in PTSD between Male and Female Refugees

Seven of the studies carried out a differential analysis of the presence of PTSD symptoms among male and female refugees (1, 3, 4, 6, 9, 10, and 12). Similar results were obtained in all of them. In general, the women were more exposed to traumatic events and scored higher in psychological problems, including PTSD, than men. For example, study 6 [54] found that 75% of female refugees had PTSD compared with 25% of the men. Furthermore, women obtained higher scores than men in intrusive symptoms, evasion symptoms, hyper-activation symptoms and overall PTSD severity. Along these same lines are the results of study 9 [57], which pointed out that the likelihood of having PTSD is much higher in women than in men. On the other hand, studies 1 [49], 4 [52] and 10 [58] showed that, even although there were no differences in the presence of PTSD among men and women, the scores for the latter were higher. These higher scores in women for PTSD also seem to become exacerbated over time. According to study 3 [51], the scores for PTSD severity were higher in women than in men at the three moments of assessment (baseline, after one year, and after three and a half years) but the differences were only statistically significant at the three-and-a-half-year follow-up.

At the same time, with regard to traumatic events, study 5 [53] analyzed the differences between a traumatic event experienced firsthand and one that was witnessed. The traumatic self-experienced event referred to whether the participant had been the victim of a traumatic event and witnessed ones, to whether the participant was the witness who observed the traumatic event involving another person. The results indicated that the number of traumatic self-experienced events significantly predicted the severity of PTSD. Furthermore, study 6 [54] revealed that the experienced traumatic load made up the most significant contributing factor to PTSD and that it was higher in refugee women.

Study 10 [58], on the other hand, assessed the effect of two types of trauma: interpersonal and non-interpersonal. The first type is conceived as a traumatic event that is perpetrated by another person with the intention of harming or threatening an individual; for example, rape or sexual abuse, torture, brainwashing, imprisonment, witnessing the murder of family members or friends, etc. Non-interpersonal trauma is not based on a relationship with others, and encompasses, for example, lack of food or water, being in poor health without access to medical care, lack of shelter, forced isolation, etc. The results of this study showed that in female refugees a link could be observed between PTSD symptoms and six of the eight traumatic interpersonal events: imprisonment, serious injuries, combat situations, brainwashing, rape or sexual abuse, and torture. On the contrary, for men, none of the subtypes of interpersonal trauma produced a significant effect in relation to PTSD symptoms. Three of the eight subtypes of non-interpersonal trauma did have an effect, however: lack of food or water, being near death and in poor health without access to medical care. In the case of women, none of the subtypes of non-interpersonal trauma produced significant effects in relation to PTSD symptoms.

### 3.3. Traumatic Experiences in Refugee Women: The Importance of Rape and Sexual Abuse

The importance of violence and sexual abuse in refugee women in relation to the presence of PTSD symptoms is reflected in six studies (4, 5, 6, 7, 10, and 12). In general, all of them reveal that refugee women suffer more sexual assault and forced sex than men do, and that these traumatic experiences are associated with a greater risk of having PTSD. For example, in study 4 [52], the rate of sexual violence reported by women was 39.7% compared to 23.6% in men, which significantly increased the presence of PTSD. In addition, according to study 5 [53], the firsthand traumatic event most often experienced among refugees is sexual assault (96.2% for this case). In study 7 [55], it was found that 87.2% of those surveyed reported having been subjected to some type of physical or sexual violence during their lives, of which 84.6% reported physical violence and 71.8% sexual violence. At the same time, the weighted prevalence of forced sex was 48.8% and attempted rape was 58.3%.

In study 6 [54], significant correlations were found between sexual assault and other types of violence by a family member or an acquaintance and PTSD. Furthermore, a relation was established between having had sexual contact before the age of 18 with an individual five or more years older and PTSD, with PTSD significantly higher in women than in men. Along these lines are studies 10 [58] and 12 [59], whose results showed a relation between rape, sexual abuse and exposure to sexual traumas, and PTSD symptoms in refugee women.

### 3.4. PTSD over Time

In the longitudinal study 3 [51], rates of PTSD were assessed in male as well as female refugees at three different moments: baseline, after one year and after three and a half years. At the beginning of the assessment, 76% met the diagnostic criteria of PTSD, 33% did so at one year, and at three and a half years, 24% of the subjects did. An inverse correlation was found between the global assessment of functioning (GAF) scores and PTSD severity scores. Although the severity of the PTSD symptoms diminished with time, the majority of the refugees continued having at least one or more symptoms related to traumas after three and a half years. In addition, the refugee women who did not speak the language of the host country seemed to be more vulnerable to the persistence of effects stemming from the trauma.

## 4. Discussion

Upon reviewing the existing literature to determine the relation between refugee women and PTSD, twelve studies dealing with this subject were identified. In these studies, important evidence was found for a high prevalence of PTSD in refugee women [55,56]. Furthermore, the presence of PTSD and its severity seems to be linked to other mental health problems, such as dissociative symptoms [56] and depression [52,53,55].

With respect to the differences between men and women, the majority of the studies pointed out that the incidence of PTSD is significantly higher in women than in men [54,57] Other studies reported that although there are no differences between the presence of PTSD in men and women, there are in fact differences with respect to its severity, as it is higher among the latter [49,52,58]. In addition, according to the study by Vojvoda et al. [51], these differences become sharper with time, finding a greater degree of severity in refugee women than in men three and half years after resettlement.

Likewise, women are more vulnerable to displaying PTSD symptoms from interpersonal-type traumatic experiences (for example, sexual abuse or torture), while men are more prone to suffering PTSD as a result of traumatic experiences of a non-interpersonal type (such as lack of food or lack of water) [58]. Additionally, refugees could suffer from PTSD if the violence incurred is carried out by a family member or an acquaintance, and in this sense, women experience it to a greater degree than men do [54]. At the same time, according to the study by Schalinski et al. [53], the severity of PTSD is determined depending on whether the traumatic event of the refugees is self-experienced or if, on the contrary, it is witnessed, with firsthand experience resulting in a greater degree of severity. For the authors, women are more likely to live through a traumatic experience firsthand than are men, for which the severity of PTSD is greater.

After a review of the different studies, it seems clear that the higher predominance and severity of PTSD in refugee women is related to gender-based traumatic experiences, such as rape, sexual assault and abuse, or genital mutilation, among others [52,53,54,55,58,59]. As stated by the World Health Organization (WHO), situations of conflict and displacement can exacerbate gender violence already existing in families and communities and bring about new forms of violence (for example, sexual slavery) against women and girls [60]. In this sense, the meta-analysis carried out by Tolin and Foa [28] on gender differences and the risk of suffering traumatic experiences and having PTSD, had observed that women are more likely than men to meet the criteria of PTSD, in spite of having a lower overall likelihood of having a traumatic experience. As such, in accordance with this review, men are more likely than women to experiences accidents, non-sexual assault, combat or war, fire or natural disaster, serious illness or non-specific injury. However, women are more likely than men to report sexual assault and child sexual abuse. Furthermore, according to the study by Schalinski et al. [53], the most common traumatic event experienced firsthand in refugees is sexual assault.

The results obtained from this review of the different studies are in line with the theory of situational vulnerability to trauma with respect to the symptoms of PTSD formulated by Pimlott-Kubiak and Cortina [34]. According to this theory, being a woman raises the risk of sexual victimization throughout an individual’s lifetime and this victimization increases the risk of suffering from PTSD [35]. Thus, the source of the main risk resides not in the female gender itself, but in the adjacent situation. For Pimlott-Kubiak and Cortina [34], female vulnerability to PTSD is simply a product of exposure to gender-based trauma and not to any attributes that women have.

In today’s world, the relation between gender-based traumatic experiences, the psychological difficulties of refugee women and possible intervention programs are an emerging topic of interest [61] and they are not reliable [62]. In fact, according to the UNHCR [63], sexual violence among female refugees is often unknown, due to different factors. On one hand, this is due to the social stigma and shame associated with rape that weighs upon the women, and on the other, due to fear of possible reprisals (rejection, blame, isolation, criminalization, increased gender inequality and punishment by other forms of violence, among others). Furthermore, as pointed out by Robbers and Morgan [61], it is likely that refugee women who are victims of sexual violence do not know how to act or where to go, are terrorized by their family members, and mistrust official procedures and authorities, since they might be immersed a circle of systematic violence. This leads to survivors often avoiding communication of their traumatic experiences, which makes us suppose that data on the presence of PTSD is not entirely accurate. This results in possible erroneous interpretations and unawareness of the psycho-socio healthcare needs of this population group [64]. This review sheds light on the relation between posttraumatic stress disorder and refugee women, especially if they have suffered a traumatic experience linked to sexual violence in any of its manifestations, which we hope will serve as a starting point for future work.

### Limitations and Future Research Lines

The main obstacle encountered in this study is the scant literature and lack of research on PTSD and the mental health of refugee women. This represented a challenge in gathering the most information possible, while also highlighting the need to analyze results found until now, aimed at gathering coherent data to promote research, create awareness among the general population, and promote social and healthcare actions in this group with their pronounced need for multidisciplinary care and intervention, particularly with respect to sexual violence. In this sense, the review undertaken by Robbers and Morgan [61] points out that sexual violence against refugee women is a complex public health concern, which requires a multicomponent solution and cultural sensitivity.

On the other hand, as observed in the study by Redwood-Campbell et al. [50], in which slightly more than one-fourth of the refugee women obtained a score indicating the presence of PTSD, these women can unconsciously block or feel uncomfortable about revealing intimate and painful experiences of sexual nature, due to religious and cultural issues, or they may be facing other barriers such as language or illiteracy. Due to these factors, the results of this study and other similar ones must be approached with caution, taking into account the numerous interferences that could arise in making a diagnosis of the mental health problems in refugees, particularly in women. In this sense, some of the studies included in this review [42,56] found numerous somatic ailments in women with histories of trauma. For socio-healthcare professionals, it is very important to take into account this expression of PTSD, because, in many instances, physical pain is more obvious and easier to detect than psychological issues. As various authors have pointed out [65,66,67,68], trauma, especially when it involves the body, stays in the body, and although possible associated emotions tend to be avoided, the body responds to the pain of the unconscious memory through somatization. For this reason, it is essential that the socio-healthcare teams who attend to this population group be aware of the involvement of PTSD or traumatic experiences in a patient’s physical symptoms and ailments, with the aim of ensuring effective treatment.

It would also be favorable to target the research to better assess symptoms of disassociation in refugee women, since it is a reaction to trauma that cannot be concealed. Work by Schalinski et al. [53] revealed that the self-experienced traumatic event most frequently reported is sexual assault, which was related with higher scores for PTSD severity that at the same time was diagnosed by disassociation, and for which it could be concluded that the disassociated response seems most common in individuals who have been sexually assaulted [29]. This finding invites us to direct possible future research lines to examine the presence of dissociative symptoms in refugee women, aimed at studying its relation to traumatic experiences of a sexual nature.

## 5. Conclusions

As a final conclusion, we would like to underline that mental health disorders are to a large extent determined by social and cultural factors and they must be taken into consideration for their analysis and intervention in refugees. In this sense, with respect to the differences between men and women, as established by the WHO [60] (p. 3), “gender determines the power differential and control that men and woman have over socioeconomic determinants in their lives and their mental health, their position, social condition, the way that they are treated in society and their susceptibility and exposure to specific risks to their mental health”. Accordingly, to understand the impact of traumatic experiences on mental health and deterioration of psychological wellbeing in refugee women, analysis from the perspective of gender is necessary, with attention paid to power relations between men and women, focusing on the significant emotional impact for women that these relations can entail, linked to having experienced traumatic events of sexual violence.

In a similar vein, this gender analysis has implications for intervention strategies aimed at survivors. Gender violence and sexual abuse must be a vital component in treatment programs and social reconstruction for refugees, particularly women. Thus, prevention and the response to sexual violence must include the active participation of refugee women in the design as well as in the implementation of prevention measures [61]. Prevention programs must hence be focused on increasing their empowerment, training and education in order to reduce sexual violence [69,70], involve the entire community [71] and transform gender norms, reinforcing community responsibility [72].

## Figures and Tables

**Table 1:** Main results and characteristics of selected studies.

ID- Article	Authors and Year	Sample	Origin of Study	Assessment Objective	Instruments	Results
1.	Robertson C.L., Halcon L., Savik K., Johnson D., (2006) [49]	N = 458 (M = 200; F = 258)	Somalia and South Central Ethiopia	PTSD (Trauma and Torture)	PCL-C	High levels of PTSD symptoms were found in women with many children.
2.	Reedwood-Campbell L., Thind H., Howard M., (2008) [50]	N = 85 F	Kosovo	PTSD	HTQ	A fourth of the population scored high in PTSD.
3.	Vojvoda D., Weine S.M., McGlashan T. (2008) [51]	N = 21 (M = 12; F = 9)	Bosnia	PTSD	PSS	Scores for PTSD severity were higher in women. A significant difference was observed at the three-and-a-half-year follow-up point.
4.	Johnson K., Scott J., Rughita B. (2010) [52]	N = 998 (405 M; 593 F)	Democratic Republic of Congo	PTSD (Sexual violence)	PSS-I	The results showed that 50.1% of the population met the criteria of PTSD, with the highest scores being among women, and 70.2% of them met criteria based on experiences of sexual violence, with scores being higher among women.
5.	Schalinski L., Elbert T., Schauer M. (2011) [53]	N = 53 F	Democratic Republic of Congo	PTSD and disassociation	PSS-I	Thirty-six subjects met all the criteria for PTSD and sexual assault was the most frequent traumatic event. The greater the disassociation and the higher the number of traumatic events, the greater the severity of PTSD.
6.	Ssenyonga J., Owens V., Olema D.K. (2012) [54]	N = 89 (M = 33; F = 56)	Democratic Republic of Congo	PTSD	PSD	Forty-four subjects suffered PTSD, of which 33 were women who scored higher than men in intrusion, evasion, and hyper-activation symptoms and in general severity of PTSD.
7.	Morof D.F., Sami S., Mangeni M (2014) [55]	N= 117 F	Democratic Republic of Congo and Somalia	PTSD (sexual and/or physical violence)	HTQ	Eighty-three women had PTSD symptoms (71% of the population).
8.	Schalinski I., Moran J., Schauer M… (2014) [56]	N = 50 F (PTSD = 33; NO PTSD = 17)	Far and Middle East, The Balkans, Africa and India	PTSD and Disassociation	CAPS; Shut- D; IAPS.	Patients with PTSD displayed intrusive memories, while the control group (NO PTSD) did not report having such memories. The women with the most severe PTSD symptoms displayed greater disassociation.
9.	Alpak G., Unal A. Bulbul F., (2015) [57]	N = 352 (M = 179; F = 173)	Syria and Turkey	PTSD	DSM-IV-TR	One hundred and eighteen of the participants were diagnosed with PTSD. Eleven of them suffered from acute PTSD, 105 from chronic PTSD and 2 from late onset PTSD.
10.	Haldane J, Nickerson A. (2016) [58]	N = 91 (M = 60; F = 31)	Iran, Sri Lanka; Afghanistan and Iraq	PTSD (impact of gender in interpersonal non-interpersonal traumatic experiences)	HTQ	A significant relation was found between non-interpersonal trauma and symptoms of PTSD. In women, a relation was observed between PTSD symptoms and traumatic interpersonal events, while in men the significant association was between PTSD symptoms and non-interpersonal traumatic events.
11.	Rometsch-Ogioun C., Denkinger J.K., Windthorst P., … (2018) [42]	-	Northern Iraq (Yazidí women)	Factors related with past histories of trauma.	Questionnaire designed by psychologists and psychologists	The psychological symptoms identified as particularly significant were nightmares, insomnia and depression.
12.	Mhlongo M.D., Tomita A., Thela L. (2018) [59]	N = 157	Burundi, Democratic Republic of Congo, Ghana, Mozambique, Ruanda, Uganda, Malawi and Zimbabwe.	Relation between experience of traumatic event and PTSD.	LEC; HTQ	Exposure to a higher number of traumatic events was associated with a higher likelihood to be at risk for PTSD. Exposure to sexual trauma was associated with a higher likelihood to be at risk for PTSD in women.

## Data Availability

Not applicable.
